# Supplementary material for: Barriers and Facilitators in Perioperative Antibiotic Prophylaxis: A Mixed-Methods Study in a Small Island Setting
Source: Antibiotics (Basel). 2021 Apr 19;10(4):462. doi: 10.3390/antibiotics10040462 (PMC8073237; doi:10.3390/antibiotics10040462)
Supplement: Supplementary file 1 [file antibiotics-10-00462-s001.zip › antibiotics-1190033-supplementary.pdf]

Supplementary files

Table S1 Results of the univariate and multivariate logistic regression for quality indicator 1 (indication)

Variables	Univariate		Multivariate[a]	
	OR (95% CI)	*p* value	OR (95% CI)	*p* value
**Sex**				
**Male**	*ref*			
**Female**	1.208 (0.477-3.061)	0.690		
	0.886 (0.686-1.138)	0.329		
**Body mass index**	1.062 (0.985-1.145)	0.120	1.075 (0.972-1.189)	0.159
**ASA classification**		0.976		
**1**	*ref*			
**2**	1.158 (0.320-4.195)	0.823		
**3**	0.917 (0.227-3.704)	0.903		
**4**	1.000 (0.050-19.963)	1.000		
**AZV (health insurance)**				
**Yes**	*ref*			
**No**	1.026 (0.195-5.406)	0.976		
**Renal function (GFR)[c]**	0.605 (0.211-1.741)	0.352		
**Knowledge renal function**				
**Yes**	1.432 (0.594-3.449)	0.424		
**No**	*ref*			
**Preoperative antibiotic use**				
**Yes**	1.727 (0.272-10.974)	0.562		
**No**	*ref*			
**Surgical ward**		0.054		
**ENT**	0.000 (0.000-.)	0.999		
**Neurosurgery**	0.071 (0.006-0.881)	0.039		
**Orthopaedic surgery**	0.019 (0.001-0.357)	0.008		
**Gynaecologic surgery**	0.185 (0.019-1.848)	0.151		
**General surgery**	0.472 (0.047-4.770)	0.525		
**Urologic surgery**	0.667 (0.032-14.033)	0.794		
**Cardiologic surgery**	0.000 (0.000-.)	0.999		
**Plastic surgery**	*ref*			
**Type of surgery**		0.997		
**Elective surgery**	*ref*			
**Emergency surgery**	1.056 (0.245-4.540)	0.942		
**Acute surgery**	$1.705*10^{9}$ (0.000-.)	1.000		
**Duration of procedure[d]**	1.000 (1.000-1.000)	0.120	1.000 (1.000-1.010)	0.063
**Blood loss[e]**	1.647 (0.740-3.639)	0.243		
**Use of implant**				
**Yes**	0.000 (0.000-.)	0.999		
**No**	*ref*			
**Duration of preoperative stay**	1.031 (0.991-1.073)	0.134	1.022 (0.972-1.074)	0.402

Nr. number, *CI* confidence interval, *OR* odds ratio, *ref* reference

[a] Model consists of body mass index, duration of procedure and duration of preoperative stay.

[b] OR per 10 years increase in age (per 1-year increase in age: univariate OR 0.988; 95% CI: 0.963-1.013)

[c] OR per 10 ml/min increase in GFR (per 1 ml/min increase in GFR: univariate OR 0.951; 95% CI: 0.856-1.057)

[d] OR per 10 minutes increase in duration of the surgical procedure (per 1 minute increase in duration: univariate OR 1.000; 95%CI: 1.000-1.000/multivariate OR 1.000; 95%: 1.000-1.010)

[e] OR per 100 millilitre increase in blood loss (per 1 millilitre increase in blood loss: univariate OR 1.005; 95% CI: 0.997-1.013)

Table S2 Results of the univariate and multivariate logistic regression for quality indicator 2 (agent)

**Variables**	**Univariate**		**Multivariate[a]**	
	**OR (95% CI)**	***p* value**	**OR (95% CI)**	***p* value**
**Sex**				
**Male**	*ref*			
**Female**	1.560 (0.448-5.431)	0.485		
**Age[b]**	0.505 (0.302-0.842)	0.009	0.516 (0.305-0.869)	0.013
**Body mass index**	1.018 (0.928-1.116)	0.705		
**ASA classification**		0.313		
**1**	*ref*			
**2**	0.000 (0.000-.)	0.999		
**3**	0.000 (0.000-.)	0.999		
**4**	0.000 (0.000-.)	0.999		
**AZV (health insurance)**				
**Yes**	*ref*			
**No**	0.394 (0.070-2.209)	0.290		
**Renal function (GFR)[c]**	0.860 (0.272-2.739)	0.801		
**Knowledge renal function**				
**Yes**	0.286 (0.056-1.466)	0.133	0.353 (0.059-2.123)	0.255
**No**	*ref*			
**Preoperative antibiotic use**				
**Yes**	1.036 (0.086-12.414)	0.978		
**No**	*ref*			
**Surgical ward**		0.366		
**Neurosurgery**	1.000 (0.000-.)	1.000		
**Orthopaedic surgery**	0.000 (0.000-.)	0.999		
**Gynaecologic surgery**	1.000 (0.000-.)	1.000		
**General surgery**	0.000 (0.000-.)	0.999		
**Urologic surgery**	1.000 (0.000-.)	1.000		
**Cardiologic surgery**	0.000 (0.000-.)	0.999		
**Plastic surgery**	*ref*			
**Type of surgery**		0.592		
**Elective surgery**	*ref*			
**Emergency surgery**	0.406 (0.072-2.281)	0.306		
**Acute surgery**	656286655 (0.000-.)	1.000		
**Duration of procedure[d]**	1.000 (1.000-1.000)	0.908		
**Blood loss[e]**	1.020 (0.942-1.105)	0.618		
**Use of implant**				
**Yes**	0.000 (0.000-.)	0.998		
**No**	*ref*			
**Duration of preoperative stay**	1.007 (0.971-1.044)	0.711		

Nr. number, *CI* confidence interval, *OR* odds ratio, *ref* reference

[a] Model consists of age and knowledge of renal function.

[b] OR per 10 years increase in age (per 1 year increase in age: univariate OR 0.934; 95% CI: 0.887-0.983/multivariate OR 0.936; 95%: 0.888-0.986)

[c] OR per 10 ml/min increase in GFR (per 1 ml/min increase in GFR: univariate OR 0.985; 95% CI: 0.878-1.106)

[d] OR per 10 minutes increase in duration of the surgical procedure (per 1 minute increase in duration: univariate OR 1.000; 95%CI: 1.000-1.000)

[e] OR per 100 millilitre increase in blood loss (per 1 millilitre increase in blood loss: univariate OR 1.002; 95% CI: 0.994-1.010)

Table S3 Results of the univariate and multivariate logistic regression for quality indicator 4 (timing)

**Variables**	**Univariate**		**Multivariate[a]**	
	**OR (95% CI)**	***p* value**	**OR (95% CI)**	***p* value**
**Sex**				
**Male**	*ref*			
**Female**	0.514 (0.131-2.020)	0.341		
**Age[b]**	1.842 (1.127-2.997)	0.014	1.791 (1.094-2.917)	0.020
**Body mass index**	0.959 (0.865-1.063)	0.428		
**ASA classification**		0.495		
**1**	*ref*			
**2**	0.242 (0.029-2.027)	0.191		
**3**	0.667 (0.084-5.301)	0.702		
**4**	$1.077*10^9$ (0.000-.)	1.000		
**AZV (health insurance)**				
**Yes**	*ref*			
**No**	4.615 (0.432-49.296)	0.206	2.906 (0.256-32.952)	0.389
**Renal function (GFR)[c]**	0.923 (0.027-31.503)	0.964		
**Knowledge renal function**				
**Yes**	1.733 (0.360-8.351)	0.493		
**No**	*ref*			
**Preoperative antibiotic use**				
**Yes**	0.000 (0.000-.)	1.000		
**No**	*ref*			
**Surgical ward**				
**Neurosurgery**				
**Orthopaedic surgery**				
**Gynaecologic surgery**				
**General surgery**				
**Urologic surgery**				
**Cardiologic surgery**				
**Plastic surgery**				
**Type of surgery**				
**Elective surgery**	*ref*			
**Emergency surgery**	3.167 (0.500-20.036)	0.221		
**Duration of procedure[d]**	1.000 (1.000-1.000)	0.367		
**Blood loss[e]**	0.819 (0.448-1.491)	0.649		
**Use of implant**				
**Yes**	1.385 (0.240-7.985)	0.716		
**No**	*ref*			
**Duration of preoperative stay**	0.995 (0.961-1.030)	0.770		

Nr. number, *CI* confidence interval, *OR* odds ratio, *ref* reference

[a] Model consists of age and AZV insurance.

[b] OR per 10 years increase in age (per 1-year increase in age: univariate OR 1.063; 95% CI: 1.012-1.116/multivariate OR 1.060; 95%: 1.009-1.113)

[c] OR per 10 ml/min increase in GFR (per 1 ml/min increase in GFR: univariate OR 0.992; 95% CI: 0.697-1.412)

[d] OR per 10 minutes increase in duration of the surgical procedure (per 1 minute increase in duration: univariate OR 1.000; 95%CI: 1.000-1.000)

[e] OR per 100 millilitres increase in blood loss (per 1 millilitre increase in blood loss: univariate OR 0.998; 95% CI: 0.992-1.004)

Table S4 Results of the univariate and multivariate logistic regression for quality indicator 5 (duration)

Variables	Univariate		Multivariate[a]	
	OR (95% CI)	*p* value	OR (95% CI)	*p* value
**Sex**				
**Male**	*ref*			
**Female**	0.000 (0.000-.)	0.998		
**Age[b]**	0.599 (0.319-1.127)	0.109	0.745 (0.365-1.524)	0.420
**Body mass index**	1.067 (0.935-1.218)	0.333		
**ASA classification**		1.000		
**1**	*ref*			
**2**	1.150 (0.105-12.619)	0.909		
**3**	$3.231*10^8$ (0.000-.)	0.998		
**4**	$3.231*10^8$ (0.000-.)	0.999		
**AZV (health insurance)**				
**Yes**	*ref*			
**No**	0.543 (0.053-5.625)	0.609		
**Renal function (GFR)[c]**	0.000 (0.000-.)	0.994		
**Knowledge renal function**				
**Yes**	0.000 (0.000-.)	0.998		
**No**	*ref*			
**Preoperative antibiotic use**				
**Yes**	0.000 (0.000-.)	0.999		
**No**	*ref*			
**Surgical ward**				
**Neurosurgery**				
**Orthopaedic surgery**				
**Gynaecologic surgery**				
**General surgery**				
**Urologic surgery**				
**Cardiologic surgery**				
**Plastic surgery**				
**Type of surgery**				
**Elective surgery**	*ref*			
**Emergency surgery**	$2.254*10^8$ (0.000-.)	0.999		
**Acute surgery**	$2.254*10^8$ (0.000-.)	1.000		
**Duration of procedure[d]**	0.834 (0.000-$7.536*10^{12}$)	0.990		
**Blood loss[e]**	0.905 (0.405-2.009)	0.716		
**Use of implant**				
**Yes**	0.080 (0.012-0.519)	0.008	0.111 (0.015-0.807)	0.030
**No**	*ref*			
**Duration of preoperative stay**	1.009 (0.951-1.070)	0.777		

Nr. number, *CI* confidence interval, *OR* odds ratio, *ref* reference

[a] Model consists of age and use of implant.

[b] OR per 10 years increase in age (per 1-year increase in age: univariate OR 0.950; 95% CI: 0.892-1.012/multivariate OR 0.971; 95%: 0.904-1.043)

[c] OR per 10 ml/min increase in GFR (per 1 ml/min increase in GFR: univariate OR 0.000; 95% CI: 0.000-.)

[d] OR per 10 minutes increase in duration of the surgical procedure (per 1 minute increase in duration: univariate OR 0.982; 95%CI: 0.050-19.396)

[e] OR per 100 millilitre increase in blood loss (per 1 millilitre increase in blood loss: univariate OR 0.999; 95% CI: 0.991-1.007)

Table S4 Results of the univariate and multivariate logistic regression for cumulative appropriateness

**Variables**	**Univariate**		**Multivariate[a]**	
	**OR (95% CI)**	***p* value**	**OR (95% CI)**	***p* value**
**Sex**				
**Male**	*ref*			
**Female**	0.979 (0.370-2.594)	0.967		
**Age[b]**	1.172 (0.904-1.524)	0.222	1.041 (0.776-1.384)	0.810
**Body mass index**	1.048 (0.969-1.133)	0.241	1.014 (0.928-1.107)	0.758
**ASA classification**		0.584		
**1**	*ref*			
**2**	1.929 (0.524-7.096)	0.323		
**3**	2.833 (0.655-12.263)	0.164		
**4**	$1.615*10^9$ (0.000-.)	0.999		
**AZV (health insurance)**				
**Yes**	*ref*			
**No**	2.857 (0.318-25.700)	0.349		
**Renal function (GFR)[c]**	0.644 (0.155-2.690)	0.549		
**Knowledge renal function**				
**Yes**	3.083 (1.209-7.861)	0.018		
**No**	*ref*			
**Preoperative antibiotic use**				
**Yes**	2.455 (0.261-23.127)	0.433		
**No**	*ref*			
**Surgical ward**		0.061		
**ENT**	0.000 (0.000-.)	0.999		
**Neurosurgery**	0.071 (0.006-0.881)	0.039		
**Orthopaedic surgery**	0.389 (0.032-4.796)	0.461		
**Gynaecologic surgery**	0.185 (0.019-1.848)	0.151		
**General surgery**	1.750 (0.134-22.778)	0.669		
**Urologic surgery**	0.667 (0.032-14.033)	0.794		
**Cardiologic surgery**	0.167 (0.012-2.368)	0.186		
**Plastic surgery**	*ref*			
**Type of surgery**		0.417		
**Elective surgery**	*ref*			
**Emergency surgery**	4.261 (0.498-36.481)	0.186		
**Acute surgery**	983332513 (0.000-.)	1.000		
**Duration of procedure[d]**	1.000 (1.000-1.010)	0.040		
**Blood loss[e]**	25.706 (0.088-7278.069)	0.257		
**Use of implant**				
**Yes**	1.507 (0.367-6.182)	0.569		
**No**	*ref*			
**Duration of preoperative stay**	2.739 (1.217-6.166)	0.015	2.101 (0.942-4.685)	0.070

Nr. number, *CI* confidence interval, *OR* odds ratio, *ref* reference

[a] Model consists of age, body mass index and duration of preoperative stay.

[b] OR per 10 years increase in age (per 1-year increase in age: univariate OR 1.016; 95% CI: 0.990-1.043)

[c] OR per 10 ml/min increase in GFR (per 1 ml/min increase in GFR: univariate OR 0.957; 95% CI: 0.830-1.104)

[d] OR per 10 minutes increase in duration of the surgical procedure (per 1 minute increase in duration: univariate OR 1.000; 95%CI: 1.000-1.001)

[e] OR per 100 millilitre increase in blood loss (per 1 millilitre increase in blood loss: univariate OR 1.033; 95% CI: 0.976-1.093)
